# Gold Inlay by Matrix and Solder Method

**Published:** 1911-09-15

**Authors:** M. L. Rudolph

**Affiliations:** Clarksville, Tenn.


### GOLD INLAY BY MATRIX AND SOLDER METHOD.

BY DR. M. L. RUDOLPH, OF CLARKSVILLE, TENN.

Read before the Tennessee State Dental Association, May 25th, 1911.

In presenting this special subject of the gold inlay by matrix and solder method, I do not wish to infer that it is the only method I use, or that it is superior to the several newer ways of making the gold inlay filling. I desire, however, to point out a few of the advantages found in the method.

I saw my first gold inlay nine or ten years ago at the Southern Dental Association held in Atlanta, Georgia. It was made by the late Dr. J. Y. Crawford. I believed then, as I know now, it was a forward step and a boon to the profession. It was a step worthy of a separate paper to set forth its advantages, the improvements and great good that has been accomplished since its advent. Since the first I have been very enthusiastic over the gold inlay, and this enthusiasm was the means by which I am now able to make a fairly good inlay, as my first attempt was accompanied with so many failures it was very disheartening. The common cause of failure at the time, was melting the edge of my matrix before I could flow my solder. At that time we were using No. 60 gold foil for our matrix. After burning a few holes and innumerable edges, I conceived the idea of thickening the gage and kept it up until I was using No. 38 gage, since which, I have forgotten the time I ruined a filling with the blow pipe.

I am sure there are several present who witnessed Dr. Crawford's demonstration and will remember how difficult it was, and what skill it required to make the inlay.

Briefly, to describe Dr. Crawford's method will state that he used a very thin matrix, I think about No. 60, 24 karat gold foil or leaf. After completing the matrix with pellets of wet cotton and burnishes, he would fill the matrix with any kind of gold filling material, withdraw and flow solder over it to make a solid gold inlay. Only those of you who have tried the method know how easy it was to melt the edge of the matrix when using high karat solder.

I will describe my method of filling a large crown cavity of the molar. One generally filled with amalgam. Take a generous piece of 24 karat plate 38 gage, lay over the top of the cavity and with as large burnisher as the cavity will chamber easily, press the center of the gold to the bottom of the cavity, hold tight and with a small burnisher work the gold down and towards the walls. Pay but little attention to the margins. That comes later. Now grasp the edge of the gold with a pair of plyers, lift out and with little care, as it matters little if the matrix does not fit perfectly the bottom or bowl of the cavity at this stage. Drop three or four pieces of solder in the matrix or enough to cover the bottom well, hold over the Bunson burner and it will only take 15 or 20 seconds to flow the solder. Dip in cold water, replace in cavity. You now have a solid bottom to your matrix and also reanealed for the second burnishing with no further danger of tearing a hole. You can now use a sharp and small instrument to hold the matrix in place while burnishing. This second burnishing should get pretty fair fitting. Lift out and trim excess to near the margins. Now fill the matrix two-thirds full of solder, replace for final burnishing. Right here is where the first particular care begins and it is absolutely necessary for success. After burnishing thoroughly around the margins, remove carefully without springing the edges of the matrix, cut a hole in the charcoal block, set the filling bowl down and finish with a blow pipe. Finish by using one piece of solder at a time. Do not put more solder than is needed, for this requires too much time to cut it out. Use just enough to allow for polishing. Your filling is always ready for a try in. It is never necessary to use whiting as the solder should never come quite to the edge of the matrix

It is no trick to fill the matrix two-thirds full of 18 karat solder and finish with 20 or 22 karat solder. The flame being directed against the higher karat it will flow before the mass is molten enough to mix.

A filling like this can be made and finished in thirty minutes or less, or a fraction longer than for an amalgam filling. Whenever it is necessary to anchor a filling or abutment for a bridge with a pin, the matrix and solder method is the simplest, surest and better way. You will have the greatest strength and absolute certainty of a perfect fit. It is not possible to make a better filling.

I am enthusiastic over the gold inlay because of the time I can and have saved. I am enabled to dispense with the rubber dam—the great friend and one cause of pyorrhea. This filling has taken the place of amalgam to a large extent in my practice. The measure of success is the results we get, and in conclusion I feel that I have accomplished more good and made more money from the gold inlay than any other one thing which has come my way in twenty-five years of actual practice.

### DISCUSSION.

Dr. R. D. Crutcher: I appreciate listening to this paper of Dr. Rudolph's as to the making of the inlay. The first inlay that I ever saw was about fourteen years ago. I have been making inlays all the time since then until the last three or four years, and I made the inlay in the way the Doctor has described it. In certain cavities that was a very good method, but, the time is past for making that kind of an inlay. The Doctor may be more skillful than some of the balance of us in saving time and in his marginal edges, etc., and so on, but I find that when we go into the proximal cavities, that it is a very difficult matter with all our skill to make these inlays in the old way as skillfully as they can be made with the casting method. I have seen some very beautiful work made in the old way, and I have seen work that has been standing for quite a number of years and is as good as when it was put in the teeth, but at the same time I have seen work that has been done in the last few years, by the casting method which is as pretty as can be made by any method. I have also seen work made by the casting apparatus that it is impossible to make in the old way, and therefore, I have laid aside the making of the soldered inlay. I never attempt to make an inlay except by the casting process as I find I can save time. Dr. Rudolph thinks that he can save time by the old way. It is true it is possible in some cases to make an inlay in the old way and possibly to save time, but I have never been successful in that mode of making an inlay, in which I didn't have to lose a great deal of time in grinding the inlay to get the surplus solder off, it is almost impossible to get just what you want and if you don't mind, if you don't get a surplus of solder there, you get a place that is not full enough, you don't get a proper contour and make the inlay as available as you can by the casting process, where you can get your molds with wax. There is almost no character of work that will present itself but what we can make the casting inlay, and for that reason I have discarded the old way of burnishing the gold into the cavity and making the inlays in that way.

Dr. Noel: I cannot see that the old method of matrix and solder has any advantage over the new process of casting inlays in the occlusal cavities of the bicuspids, and more especially the molars, where we have a box form into which the solder will flow; but where you have to make a contour it is an extremely difficult matter to flow the solder to the exposed surface of the cavity in proper shape. And where-ever you have proximal cavities in the incisors and cuspids, it is almost an impossibility to restore the contour to this tooth. The first inlay I ever made was during my senior year for an old lady, on the incisal edges of the two central incisors. I never had such a time in my life restoring the contour of those teeth, I finally got enough solder there to restore the length of the teeth, I had enough solder bellied out toward the labial to keep me grinding the half of an afternoon in the laboratory to get that solder out of the way. So I don't see any advantage to be gained.

Dr. Leonard: I use the method spoken of by Dr. Rudolph for several years with a great deal of satisfaction, and in fact I hesitated a long time to begin the casting method because I was so pleased with the other, and felt that I was getting just as good results as I could get by the casting method. I have since abandoned that method and use the casting method, because I have found more satisfaction in the use of that method perhaps. I find that I can get better results occasionally by a combination of the two methods in cases where I want to make a little hood for instance for an attachment for a bridge or for the construction of a crown by using a matirx of gold plate and flowing solder on it to stiffen it. I burnish it to fit by repeated burnishings and adjust the gold matrix and stiffening it up with solder and then by covering it with wax and carving it out in the shape I want, I think this simplifies the casting. I think probably I can do better work, and there is no doubt but that a careful operator can get splendid results and practically perfect results by the method outlined by Dr. Rudolph, and there is not any question in my mind but what it is an excellent method, but the other is simpler and as a rule more accurate, and you get better occlusion.

Dr. Rudolph: I would like to say that I have been following this method for five or six years, but for a year I don't think I have made one burnished gold inlay, but I am losing so much time that I don't think casting a good plan. I will say frankly that I do not think a gold inlay made in this way is quite as pretty as the casting, but it is simply to do away with the loss of time and to fill a cavity that you would otherwise fill by that method as I have seen it done, and then you can demand a good price for it. Now to any one who is not familiar with the method, I think it hardly worth your while to learn it. You can learn casting easier. But if you ever learn this method I don't believe you will soon give it up.

